# Angiogenesis in breast cancer: insights and innovations

**DOI:** 10.1007/s10238-024-01446-5

**Published:** 2024-08-06

**Authors:** Ghada Elayat, Abdel Selim

**Affiliations:** 1 Department of Natural Science, Middlesex University, Hendon, London, UK; 2 Pathology Department, Faculty of Medicine, Tanta University, Tanta, Egypt; 3 Histopathology Department, King’s College Hospital, Denmark Hill, London, UK

**Keywords:** Angiogenesis, Breast cancer, Biomarkers, Imaging techniques, Therapeutic approaches, Resistance mechanisms, Clinical implications, Personalized medicine

## Abstract

This review explores the pivotal role of angiogenesis in breast cancer progression and treatment. It covers biomarkers, imaging techniques, therapeutic approaches, resistance mechanisms, and clinical implications. Key topics include Vascular Endothelial Growth Factors, angiopoietins, microRNA signatures, and circulating endothelial cells as biomarkers, along with Magnetic Resonance Imaging, Computed Tomography Angiography, Ultrasound, and Positron Emission Tomography for imaging. Therapeutic strategies targeting VEGF, tyrosine kinase inhibitors, and the intersection of angiogenesis with immunotherapy are discussed. Challenges such as resistance mechanisms and personalized medicine approaches are addressed. Clinical implications, prognostic value, and the future direction of angiogenesis-targeted therapies are highlighted. The article concludes with reflections on the transformative potential of understanding angiogenesis.

## Basics of angiogenesis: unveiling the foundation of blood vessel formation

### Introduction to angiogenesis

Angiogenesis, a fundamental physiological process, serves as the intricate choreography underlying the formation of blood vessels [1]. In this foundational section, we embark on a journey to unravel the essential principles of angiogenesis, aiming to shed light on the mechanisms orchestrating the birth of new vessels [2]. Understanding the nuances of this dynamic process is paramount, as it plays a pivotal role not only in normal physiological functions but also in the context of pathological conditions, notably cancer [3].

### Dynamics of blood vessel formation

The section delves into the dynamic interplay between pro-angiogenic and anti-angiogenic factors, illuminating the delicate equilibrium that governs vascular homeostasis [4]. We explore the pivotal role of vascular endothelial growth factors (VEGFs), angiopoietins, and other signaling molecules in initiating and regulating angiogenesis [5]. This nuanced exploration lays the groundwork for comprehending the intricacies of blood vessel formation [6].

### Molecular players in angiogenesis

Moving beyond the surface, we unravel the molecular players that choreograph angiogenesis, providing a detailed insight into the signaling pathways that govern endothelial cell behavior [7]. VEGF, a linchpin in this process, takes center stage, orchestrating a cascade of events that lead to the sprouting and maturation of new blood vessels [8]. The balance between these pro-angiogenic forces and the counteracting anti-angiogenic signals paints a comprehensive picture of the regulatory mechanisms at play [9].

### Implications for normal physiology

While angiogenesis is essential for embryonic development, wound healing, and tissue repair in normal physiological contexts, the section examines how this process goes awry in pathological conditions [10]. The dysregulation of angiogenesis is a hallmark of various diseases, and a deeper understanding of its basics forms the foundation for unraveling its implications in the subsequent sections, notably its role in the context of breast cancer [11].

As we embark on this exploration of the basics of angiogenesis, the aim is not only to elucidate the molecular intricacies but also to set the stage for a comprehensive understanding of how this foundational process becomes a double-edged sword in the context of cancer, particularly breast cancer [6]. The insights gained here will serve as a scaffold for navigating the complexities of blood vessel formation within the context of malignancy in the sections that follow [12].

## Angiogenesis in breast cancer

### Overview of dysregulation in breast cancer

Angiogenesis, the formation of new blood vessels from existing ones, is a tightly regulated process crucial for normal tissue development and repair [1]. However, in the context of breast cancer, this finely tuned mechanism becomes dysregulated, propelling the tumour into a state of uncontrolled growth and progression [2]. The tumour microenvironment, rich in growth factors and cytokines, fosters an environment conducive to angiogenesis, ensuring a continuous supply of nutrients and oxygen to sustain the growing tumour mass [3].

In breast cancer, dysregulation of angiogenesis is often orchestrated by an imbalance between pro-angiogenic and anti-angiogenic factors [4]. The tumour cells, stimulated by hypoxia and other microenvironmental cues, release a cascade of signaling molecules, prominently vascular endothelial growth factors (VEGFs) and angiopoietins [5]. These molecules act on endothelial cells, triggering a series of events that culminate in the sprouting of new blood vessels, a process fundamental to tumour survival and progression [6].

### Relationship between tumour growth and blood supply

The relationship between tumour growth and angiogenesis is symbiotic [7]. As breast tumours grow beyond a critical size, they outstrip their existing blood supply, leading to regions of hypoxia [8]. This hypoxic microenvironment serves as a potent stimulus for angiogenesis, prompting the tumour cells to release angiogenic factors [9]. In response, new blood vessels sprout and infiltrate the tumour, establishing a network that not only provides essential nutrients and oxygen but also facilitates the dissemination of cancer cells to distant sites, a process crucial for metastasis [10].

The molecular crosstalk between tumour cells and endothelial cells plays a pivotal role in shaping the angiogenic phenotype of breast cancer [11]. The release of pro-angiogenic factors by tumour cells activates endothelial cells, initiating a cascade of events that involves the degradation of the extracellular matrix, endothelial cell migration, and the formation of new blood vessels [6]. This intricate dance between tumour cells and the vascular network highlights the dynamic and adaptive nature of angiogenesis in breast cancer [12].

### The role of hypoxia and the tumour microenvironment

Hypoxia, or low oxygen levels, is a hallmark feature of the tumour microenvironment and a potent inducer of angiogenesis in breast cancer [12]. As tumours rapidly proliferate, they outgrow their existing blood supply, creating regions of hypoxia within the tumour mass [8]. In response to hypoxia, tumour cells activate hypoxia-inducible factors (HIFs), transcription factors that orchestrate the expression of various pro-angiogenic genes, including VEGF [13].

The HIF-VEGF axis represents a critical molecular pathway driving angiogenesis in breast cancer [12]. VEGF, the master regulator of angiogenesis, acts on endothelial cells, stimulating their proliferation and migration [14]. Additionally, hypoxia-induced factors contribute to the recruitment of endothelial progenitor cells from the bone marrow, further fueling the angiogenic process [15]. The adaptation of breast cancer cells to thrive in hypoxic conditions underscores the dynamic nature of tumour angiogenesis and the intricate interplay between oxygen sensing and vascularization [12].

### Molecular mechanisms involved in angiogenesis in breast cancer

The molecular landscape of angiogenesis in breast cancer is intricate, involving a plethora of signaling pathways and molecular players [3]. VEGF, a family of growth factors, stands as a central orchestrator, binding to its receptors on endothelial cells and initiating a cascade of events leading to blood vessel formation [16]. The angiopoietin-Tie2 pathway, another key player, contributes to the stabilization and maturation of newly formed blood vessels [17].

Beyond these, other signaling pathways, including fibroblast growth factor (FGF), platelet-derived growth factor (PDGF), and transforming growth factor-beta (TGF-β), intricately weave into the angiogenic tapestry of breast cancer [18]. These pathways not only influence endothelial cell behavior but also contribute to the complex interplay between the tumour and its microenvironment, shaping the angiogenic phenotype of breast cancer [19].

Understanding these molecular mechanisms is essential for the development of targeted therapies aimed at disrupting angiogenesis in breast cancer [7]. As research progresses, novel targets and therapeutic strategies are continually being identified, opening avenues for precision medicine and personalized approaches tailored to the specific molecular makeup of individual breast tumours [20]. The unraveling of these intricate molecular pathways not only enhances our understanding of breast cancer biology but also provides a roadmap for the development of innovative therapeutic interventions aimed at curbing the angiogenic drive of this formidable disease [18].

## Biomarkers and imaging in angiogenesis research

### Biomarkers: unraveling the molecular signature of angiogenesis in breast cancer

As our understanding of angiogenesis in breast cancer deepens, the identification and validation of biomarkers have become paramount in both research and clinical settings [3]. Biomarkers serve as measurable indicators of biological processes and can play a pivotal role in diagnosing, prognosticating, and predicting the response to treatment. In the context of angiogenesis, researchers have fervently sought biomarkers that mirror the dynamic changes occurring in the tumour microenvironment.

- Vascular endothelial growth factors (VEGFs): Among the myriad of biomarkers, VEGFs take center stage [8]. These signaling proteins play a pivotal role in stimulating the formation of new blood vessels. In breast cancer, VEGF-A, in particular, has been extensively studied for its association with tumour angiogenesis (9). Elevated levels of VEGF-A are often correlated with increased microvessel density within tumours, signifying a pro-angiogenic milieu that fuels tumour growth.
- Angiopoietins and tie receptors: Beyond VEGFs, angiopoietins, and their cognate receptors, particularly Tie-2, contribute to the fine-tuning of angiogenesis [17]. Dysregulation of angiopoietin-Tie signaling has been implicated in the pathogenesis of breast cancer, and their assessment as potential biomarkers provides a multifaceted view of the angiogenic landscape.
- MicroRNA signatures: The realm of non-coding RNA has unveiled microRNAs as potential regulators of angiogenesis [3]. Specific microRNA signatures associated with angiogenesis in breast cancer have been identified, offering a novel avenue for diagnostic and prognostic applications. These small RNA molecules intricately modulate gene expression, influencing the delicate balance between pro- and anti-angiogenic factors.
- Circulating endothelial cells and circulating endothelial progenitor cells: The quest for non-invasive biomarkers has led researchers to explore circulating endothelial cells (CECs) and circulating endothelial progenitor cells (CEPs) [15]. These cells, shed from actively growing blood vessels, can be quantified in peripheral blood, providing a dynamic snapshot of angiogenic activity. The enumeration and characterization of CECs and CEPs hold promise as biomarkers for monitoring disease progression and assessing treatment response.

### Imaging techniques: unveiling the vascular architecture of breast tumours

In tandem with biomarker research, advancements in imaging technologies have revolutionized our ability to visualize the intricate vascular architecture within breast tumours [19]. Non-invasive imaging modalities offer clinicians and researchers unprecedented insights into the spatial and temporal dynamics of angiogenesis, facilitating early detection, accurate staging, and monitoring of therapeutic responses.

- Magnetic resonance imaging (MRI): MRI, with its high soft tissue contrast, has emerged as a powerful tool for visualizing angiogenesis in breast cancer. Dynamic contrast-enhanced MRI (DCE-MRI) allows for real-time assessment of contrast agent kinetics, enabling the characterization of tumour vasculature and perfusion [8]. This modality aids in distinguishing between benign and malignant lesions and provides valuable information for treatment planning.
- Computed tomography (ct) angiography: CT angiography provides detailed three-dimensional images of blood vessels [11], allowing for the visualization of abnormal angiogenesis within breast tumours. This modality is particularly valuable for assessing vascularization patterns, detecting microcalcifications, and aiding in the differentiation of benign and malignant lesions.
- Ultrasound imaging: Doppler ultrasound, a cost-effective and widely available imaging modality, allows for the assessment of blood flow within breast tumours [10]. Color Doppler and power Doppler techniques provide valuable information on vascularity, aiding in the characterization of lesions and guiding biopsy procedures.
- Positron emission tomography (PET): PET imaging, coupled with radiotracers targeting angiogenesis-related molecules, offers a functional perspective on tumour vascularity [7]. The use of radiotracers such as 18F-fluorodeoxyglucose (FDG) or novel agents specifically targeting angiogenic markers allows for the quantitative assessment of angiogenesis within breast tumours.

### Future directions in biomarker and imaging research

The integration of biomarkers and advanced imaging techniques holds tremendous potential in refining our understanding of angiogenesis in breast cancer [4]. Future research endeavors should focus on the discovery of novel biomarkers with higher specificity and sensitivity, allowing for more accurate disease stratification and prediction of treatment response [12]. Moreover, the development of multimodal imaging approaches, combining the strengths of different techniques, promises a more comprehensive assessment of angiogenic activity within tumours.

As we progress into an era of personalized medicine, the identification of patient-specific angiogenic profiles and the incorporation of these profiles into treatment algorithms could revolutionize therapeutic decision-making [20]. The journey toward unraveling the intricacies of angiogenesis in breast cancer continues, guided by the dual beacons of molecular insights and technological innovations. Through the collaborative efforts of researchers and clinicians, the integration of biomarkers and imaging modalities is poised to usher in a new era of precision oncology, where the integration of biomarkers and imaging modalities is poised to usher in a new era of precision oncology, where the battle against breast cancer is fought with unprecedented accuracy and efficacy.

## Therapeutic approaches targeting angiogenesis

### Overview of anti-angiogenic therapies

The recognition of angiogenesis as a hallmark of tumour growth has prompted a paradigm shift in cancer therapeutics [18]. In the context of breast cancer, anti-angiogenic therapies have emerged as a promising avenue to impede tumour progression. These therapeutic strategies aim to disrupt the intricate vascular network supplying nutrients and oxygen to the tumour, thereby stalling its growth and metastatic potential.

One of the key players in anti-angiogenic therapy is the inhibition of vascular endothelial growth factors (VEGF), which play a central role in stimulating the formation of new blood vessels [16]. Several anti-VEGF agents, such as bevacizumab, have been at the forefront of clinical trials and have gained approval for use in breast cancer. These agents function by binding to VEGF, preventing its interaction with its receptors on endothelial cells and thwarting the angiogenic cascade.

### Discussion of currently approved drugs

Bevacizumab, a monoclonal antibody targeting VEGF, stands as a pioneer in anti-angiogenic therapy for breast cancer [16]. Approved for use in combination with chemotherapy, bevacizumab has demonstrated efficacy in certain subsets of breast cancer patients, particularly in the metastatic setting. However, the landscape of anti-angiogenic therapies is continually evolving, with ongoing research focusing on refining existing approaches and identifying novel targets.

Tyrosine kinase inhibitors (TKIs), such as sunitinib and sorafenib, represent another class of drugs employed in anti-angiogenic strategies [16]. These small molecules interfere with signaling pathways crucial for angiogenesis by targeting receptors involved in the process. While these agents have shown promise in preclinical studies and some clinical trials, challenges persist, including the development of resistance and adverse effects.

### Challenges and future directions

Despite the initial enthusiasm surrounding anti-angiogenic therapies, several challenges impede their widespread success in breast cancer treatment [8]. One significant hurdle is the development of resistance mechanisms within the tumour microenvironment. Tumours, characterized by their adaptive nature, can activate alternative angiogenic pathways or undergo phenotypic changes that render them less susceptible to anti-angiogenic agents.

Understanding and overcoming these resistance mechanisms are critical for enhancing the efficacy of anti-angiogenic therapies [12]. Combinatorial approaches, involving the simultaneous targeting of multiple angiogenic pathways or the integration of anti-angiogenic agents with immunotherapy, are actively being explored to address these challenges. Moreover, identifying predictive biomarkers that can stratify patients based on their likelihood of responding to anti-angiogenic therapies is a pressing need in the field.

### Potential strategies to overcome resistance

In the pursuit of overcoming resistance to anti-angiogenic therapies, researchers are investigating a spectrum of innovative strategies [11]. Combination therapies, incorporating anti-angiogenic agents with traditional chemotherapy or immunotherapy, aim to capitalize on synergistic effects that could enhance treatment outcomes. Additionally, efforts are underway to identify and target specific molecular alterations within tumours that confer resistance, allowing for more precise and personalized therapeutic interventions.

The integration of biomarker-driven approaches holds promise in refining patient selection for anti-angiogenic therapies [20]. Biomarkers indicative of a tumour’s angiogenic profile or its propensity to develop resistance could guide clinicians in tailoring treatment regimens. Furthermore, advancements in imaging technologies allow for real-time monitoring of angiogenic responses, facilitating adaptive treatment strategies that can be modified based on the evolving nature of the tumour.

### The intersection of angiogenesis and immunotherapy

Recent advances in cancer immunotherapy have opened new avenues for synergy with anti-angiogenic approaches [7]. The intricate crosstalk between the immune system and angiogenesis is being explored to develop combinatorial strategies that harness the body's immune response while simultaneously targeting the tumour vasculature. Immune checkpoint inhibitors, such as PD-1 and PD-L1 inhibitors, are being investigated in combination with anti-angiogenic agents, aiming to amplify the anti-tumour immune response.

The evolving landscape of anti-angiogenic therapies in breast cancer underscores the dynamic nature of cancer research and treatment [10]. The interplay between angiogenesis, the tumour microenvironment, and the immune system offers a multifaceted approach to therapeutic intervention. As we delve into the complexities of these interactions, the next frontier in breast cancer therapeutics may well lie in the convergence of anti-angiogenic strategies with immunotherapy, heralding a new era of precision medicine tailored to the unique biology of each patient's tumour.

## Resistance to anti-angiogenic therapies

### Mechanisms leading to resistance

The emergence of resistance to anti-angiogenic therapies represents a significant obstacle in their clinical efficacy [11]. Tumours, inherently adaptive entities, can evolve to circumvent the inhibitory effects of these therapies, leading to treatment resistance. One primary mechanism involves the upregulation of alternative pro-angiogenic pathways [7]. In response to the inhibition of a specific pathway, tumours may activate compensatory mechanisms, such as the fibroblast growth factor (FGF) pathway or the angiopoietin-Tie2 axis, to sustain angiogenesis.

Another avenue through which tumours develop resistance is through the recruitment of bone marrow-derived cells, such as endothelial progenitor cells, to compensate for the inhibited angiogenesis [8]. This intricate interplay between the tumour and its microenvironment highlights the dynamic nature of resistance mechanisms, necessitating a comprehensive understanding to devise effective counterstrategies.

### Adaptive changes in tumours

Tumours possess a remarkable ability to adapt and evolve under selective pressures imposed by therapeutic interventions [10]. Anti-angiogenic therapies create a hostile microenvironment for tumour cells by compromising their blood supply, leading to hypoxia and nutrient deprivation. In response, tumours may undergo phenotypic changes, becoming more invasive and resistant to apoptosis. This adaptation often involves the activation of survival pathways, such as the phosphoinositide 3-kinase (PI3K)/Akt/mammalian target of rapamycin (mTOR) pathway, to sustain growth despite the hostile conditions induced by anti-angiogenic agents.

Additionally, the tumour microenvironment undergoes remodeling, promoting the selection of more aggressive and treatment-resistant cell populations. The emergence of these subpopulations further complicates therapeutic strategies, emphasizing the need for a holistic understanding of the evolving tumour landscape during anti-angiogenic treatment.

### Potential strategies to overcome resistance

Addressing resistance to anti-angiogenic therapies requires a multifaceted approach that considers the intricate biology of both the tumour and its microenvironment [11]. Combination therapies that target multiple angiogenic pathways simultaneously or integrate anti-angiogenic agents with other modalities, such as chemotherapy or immunotherapy, present promising avenues. This combinatorial approach seeks to create a more comprehensive blockade against pro-angiogenic signals, minimizing the chances of tumours activating alternative pathways.

Moreover, efforts are underway to identify predictive biomarkers that can stratify patients based on their susceptibility to developing resistance [20]. Biomarkers associated with the activation of compensatory pathways or the presence of specific genetic alterations may guide clinicians in tailoring treatment regimens for enhanced efficacy.

The concept of adaptive treatment strategies is gaining traction in the realm of anti-angiogenic therapies. Real-time monitoring of treatment responses through advanced imaging techniques allows clinicians to adapt therapeutic interventions based on the evolving dynamics of the tumour. This dynamic approach acknowledges the heterogeneity and adaptability of tumours, aiming to stay one step ahead in the ongoing battle against resistance.

### Personalized medicine and resistance

The era of personalized medicine is gradually reshaping the landscape of cancer treatment, and anti-angiogenic therapies are no exception [3]. Understanding the genetic and molecular profile of individual tumours becomes imperative in devising personalized treatment regimens that account for the unique characteristics of each patient's cancer. Advances in genomic profiling and molecular diagnostics enable the identification of specific mutations or alterations that may influence the response to anti-angiogenic agents.

Tailoring treatment based on the genetic makeup of tumours holds the potential to overcome resistance by addressing the specific vulnerabilities of each cancer [10]. This approach not only optimizes therapeutic outcomes but also minimizes unnecessary exposure to treatments that may be futile due to inherent resistance mechanisms.

As we navigate the intricate challenges posed by resistance to anti-angiogenic therapies, a comprehensive understanding of the dynamic interplay between tumours and their microenvironment is essential. By embracing a multifaceted strategy that combines molecular insights, adaptive treatment approaches, and personalized medicine, the field aims to turn the tide against resistance, fostering a new era of more effective and sustainable anti-angiogenic therapies for breast cancer patients.

## Clinical implications and prognostic value

### Prognostic value of angiogenesis in breast cancer

The assessment of angiogenesis in breast cancer holds profound prognostic implications, serving as a critical determinant of tumour behavior and patient outcomes [3]. Extensive research has illuminated the correlation between increased microvessel density, a surrogate marker for angiogenesis, and adverse prognostic factors in breast cancer. Tumours exhibiting heightened angiogenesis often manifest increased invasiveness, lymph node involvement, and a higher likelihood of distant metastasis.

Moreover, the degree of angiogenesis within a breast tumour has been linked to overall survival and disease-free survival [10]. Patients with tumours characterized by robust angiogenesis generally face a less favorable prognosis, underlining the pivotal role of vascularization in the aggressive phenotype of certain breast cancers. Integrating angiogenic assessments into standard prognostic models enhances the precision of outcome predictions, aiding clinicians in tailoring treatment strategies based on the vascular landscape of the tumour.

### Clinical implications of targeting angiogenesis

The translation of angiogenesis research into clinical practice has given rise to a paradigm shift in the management of breast cancer [3]. Targeting angiogenesis represents a therapeutic avenue with profound clinical implications, offering a complementary strategy to conventional treatments. Anti-angiogenic agents, such as bevacizumab, have found their way into the clinical armamentarium, particularly in the metastatic setting, where they demonstrate efficacy in curtailing tumour progression and improving progression-free survival.

In addition to their standalone role, anti-angiogenic therapies are increasingly being explored in combination with traditional chemotherapy regimens, radiotherapy, and immunotherapy. Combinatorial approaches aim to capitalize on synergistic effects, providing a comprehensive assault on the tumour microenvironment. However, the clinical landscape is dynamic, and ongoing research endeavors continue to refine the integration of anti-angiogenic agents into treatment algorithms, with a keen eye on optimizing therapeutic outcomes while mitigating potential side effects.

### Review of clinical trials

The evaluation of anti-angiogenic therapies through rigorous clinical trials constitutes a cornerstone in deciphering their true clinical impact [3]. Numerous trials have investigated the efficacy of these agents in different settings of breast cancer, spanning from the neoadjuvant and adjuvant settings to metastatic disease. While some trials have demonstrated promising results, others have faced challenges, including issues related to patient selection, the optimal timing of intervention, and the identification of predictive biomarkers.

Through systematic review and meta-analysis, researchers seek to distill the collective evidence from clinical trials, providing a comprehensive overview of the impact of anti-angiogenic therapies on patient outcomes [10]. This scrutiny aids in discerning patterns of efficacy, identifying patient subgroups that stand to benefit the most, and refining the criteria for selecting candidates who are likely to respond favorably to these interventions.

### Challenges and future directions

Despite the progress made in understanding angiogenesis and harnessing it for therapeutic purposes, challenges persist on the road to clinical success [8]. The identification of robust predictive biomarkers remains a pressing need, allowing for the stratification of patients based on their likelihood of responding to anti-angiogenic therapies. Additionally, the intricate interplay between the tumour microenvironment, immune system, and angiogenesis necessitates a more nuanced understanding to optimize combinatorial treatment strategies.

Future directions in the clinical realm involve the exploration of novel agents and innovative therapeutic modalities [10]. The convergence of angiogenesis-targeting approaches with immunotherapy, for instance, represents an exciting frontier with the potential to revolutionize breast cancer treatment. Harnessing the immune system's ability to recognize and eliminate tumour cells, in conjunction with disrupting angiogenesis, offers a synergistic approach that may overcome existing challenges and elevate therapeutic outcomes.

## Conclusion

In the quest to understand and combat breast cancer, this review has traversed the intricate landscape of angiogenesis, highlighting its pivotal role in tumour progression. Angiogenesis, a process co-opted by cancer cells to sustain growth, has been dissected at the molecular level, revealing promising avenues for therapeutic intervention.

The integration of anti-angiogenic therapies marks a paradigm shift in breast cancer treatment, yet challenges like resistance mechanisms persist. However, advancements in combinatorial approaches and personalized medicine offer hope for improved outcomes.

As we synthesize knowledge from bench to bedside, the clinical implications of angiogenesis in breast cancer management become increasingly evident. This review underscores the transformative potential of understanding angiogenesis, inspiring ongoing efforts to innovate and improve patient care.

In conclusion, the journey through the angiogenic landscape of breast cancer illuminates not only current challenges but also future opportunities. The relentless pursuit of knowledge and innovation promises to reshape the trajectory of breast cancer care, offering hope for those affected by this disease.

## Data Availability

No datasets were generated or analyzed during the current study.
